# 159. Improving Antibiotic Use for Sinusitis and Upper Respiratory Tract Infections: A Virtual Visit Antibiotic Stewardship Initiative

**DOI:** 10.1093/ofid/ofab466.159

**Published:** 2021-12-04

**Authors:** Anastasia Wasylyshyn, Keith S Kaye, Julia Chen, Haley Haddad, Jerod Nagel, Joshua G Petrie, Tejal N Gandhi, Lindsay A Petty

**Affiliations:** 1 University of Michigan, Ann Arbor, Michigan; 2 University of Michigan Medical School, Ann Arbor, MI

## Abstract

**Background:**

Asynchronous virtual patient care is growing in popularity; however, the effectiveness of virtually delivering guideline-concordant care in conjunction with antibiotic stewardship initiatives remains uncertain. We developed a bundled stewardship intervention aimed at improving antibiotic use in E-visits for upper respiratory tract infections (URTIs).

**Methods:**

In this pre-post study, adult patients who completed an E-visit for “cough,” “flu,” or “sinus symptoms” at Michigan Medicine between 1/1/2018 and 9/30/2020 were included. Patient demographics, diagnoses, and antibiotic details were collected. The multi-faceted intervention occurred over 6 months (Figure 1).

We performed segmented linear regression to estimate the effect of the intervention on the level and trend of appropriate antibiotic use for URTI diagnosis (defined as no antibiotic prescribed) and sinusitis (defined as guideline-concordant antibiotic selection and duration). Regression lines were fit to data before (March 2019) and after (May 2019) the physician championing period.

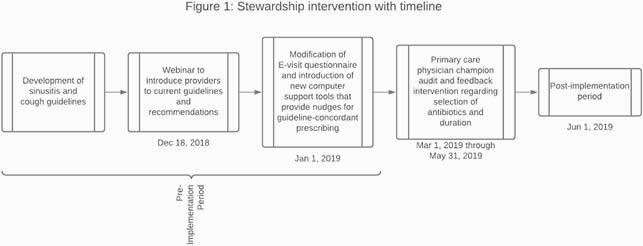

**Results:**

Among 5151 E-visits, the mean age was 46 years old, and most patients were female (71.3%, N=3674). 3405/5151 E-visits were for URTI. Inappropriate antibiotic use for URTI was stable in trend prior to the audit and feedback intervention (Figure 2), followed by a 12% (P-value = 0.01) decrease in inappropriate antibiotic use post-intervention. The trend in inappropriate antibiotic use continued to decrease after the intervention by 1.1%/month (P-value = 0.02) (Figure 2a).

Of 2493/5151 E-visits specifically for sinus symptoms, guideline-concordant antibiotic use was low (intercept = 8%) pre-intervention (Figure 2b). Post-intervention, there was an estimated 47% increase (P-value < 0.001) in patients receiving guideline-concordant antibiotics.

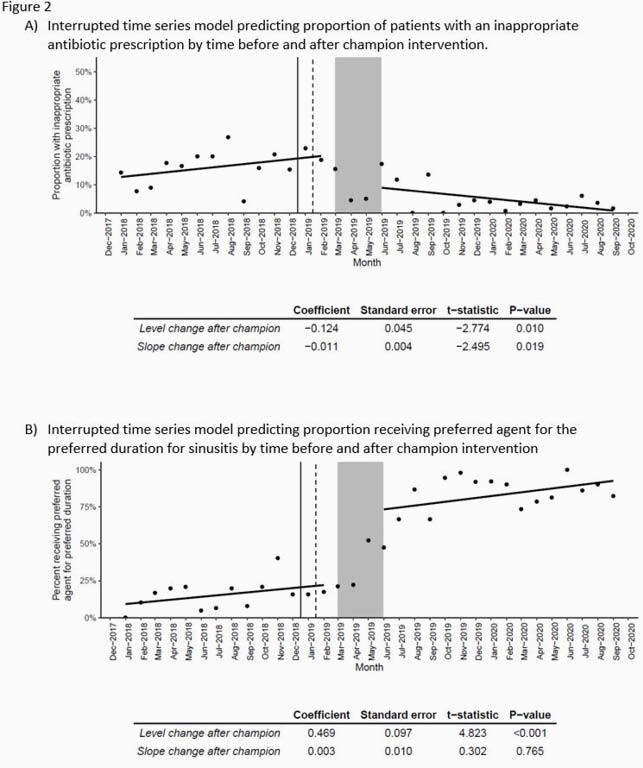

Solid line represents time of the webinar, dashed line represents time of modified questionnaire roll out and electronic medical record “nudges”, and shaded area is time of physician champion intervention. Guideline-concordant antibiotic prescribing for sinusitis included amoxicillin/clavulanate or doxycycline prescribed for a duration of 5-7 days

**Conclusion:**

A multifaceted stewardship bundle for E-visits improved guideline-concordant antibiotic use for URTIs. Changes implemented in the EMR are most beneficial after a period of audit and feedback. This approach can aid stewardship efforts in the ambulatory care setting particularly with regards to telemedicine.

**Disclosures:**

**Tejal N. Gandhi, MD**, Blue Cross Blue Shield of Michigan (Individual(s) Involved: Self): Grant/Research Support **Lindsay A. Petty, MD**, Nothing to disclose

